# Community Synergy of Lactic Acid Bacteria and Cleaner Fermentation of Oat Silage Prepared with a Multispecies Microbial Inoculant

**DOI:** 10.1128/spectrum.00705-23

**Published:** 2023-05-11

**Authors:** Lin Sun, Yanlin Xue, Yanzi Xiao, Rigele Te, Xiaoguang Wu, Na Na, Nier Wu, Moge Qili, Yi Zhao, Yimin Cai

**Affiliations:** a Inner Mongolia Engineering Research Center of Development and Utilization of Microbial Resources in Silage, Inner Mongolia Academy of Agriculture and Animal Husbandry Sciences, Hohhot, Inner Mongolia, People’s Republic of China; b College of Agriculture and Forestry, Hulunbuir University, Hulunber, Inner Mongolia, People’s Republic of China; c Inner Mongolia Autonomous Region Land Surveying and Planning Institute, Hohhot, Inner Mongolia, People’s Republic of China; d Japan International Research Center for Agricultural Sciences, Tsukuba, Ibaraki, Japan; University of Torino

**Keywords:** clean fermentation, community synergy, gas production, lactic acid bacteria, oat

## Abstract

To investigate community synergy of lactic acid bacteria (LAB) and cleaner fermentation of oat silage, oat silages were prepared with or without (control) commercial LAB inoculants LI1 (containing Lactiplantibacillus plantarum, Lentilactobacillus buchneri, Lacticaseibacillus paracasei, and Pediococcus acidilactici) and LI2 (containing *Lactiplantibacillus plantarum* and Lentilactobacillus buchneri). The microbial community, LAB synergy, and cleaner fermentation were analyzed at 1, 3, 6, 15, 35, and 90 days of ensiling. The LAB inoculant improved fermentation quality, with significantly (*P < *0.05) lower pH, ammonia nitrogen content, and gas production and higher lactic acid and acetic acid contents than those of the control. *Enterobacteriaceae* was the main bacterial community in early stage of fermentation, which utilizes sugar to produce CO_2_ gas, causing dry matter (DM) and energy loss. As fermentation progressed, the microbial diversity decreased, and the microbial community shifted from Gram-negative to Gram-positive bacteria. The inoculation of multispecies LAB displayed community synergy; Pediococcus acidilactici formed a dominant community in the early stage of fermentation, which produced an acid and anaerobic environment for the subsequent growth of *Lentilactobacillus* and *Lacticaseibacillus* species, thus forming a LAB-dominated microbial community. The predicted functional profile indicated that the silage inoculated with LI1 enhanced the carbohydrate metabolism pathway but inhibited the amino acid metabolism pathway, which played a role in promoting faster lactic acid production, reducing the decomposition of protein to ammonia nitrogen, and improving the fermentation quality of silage. Therefore, oat silage can be processed to high-quality and cleaner fermented feed by using an LAB inoculant, and LI1 showed better efficiency than LI2.

**IMPORTANCE** Oat natural silage is rich in *Enterobacteriaceae*, increasing gas production and fermentation loss. Lactic acid bacteria interact synergistically to form a dominant community during ensiling. Pediococci grow vigorously in the early stage of fermentation and create an anaerobic environment. Lactobacilli inhibit the harmful microorganisms and result in cleaner fermentation of oat silage.

## INTRODUCTION

Oat (Avena sativa) is a dual-purpose crop, widely planted in Europe, North America, and northern China for food and feed production (1). Livestock production in many countries around the world is plagued by feed shortages during cold winter and early spring, increasing the demand for good-quality oat production (2). However, high temperatures and rain during the harvest period often delay the harvest, resulting in high levels of crop dry matter (DM) and fiber contents and losses of nutrients during the processing and storing of oat hay (3). Therefore, oat needs to be appropriately stored to ensure provision throughout the year of good-quality forage for herbivores. Silage preservation is crucial for maintaining forage quality and alleviating seasonal and regional imbalances in quality forage supply. Recently, the study of oat silage has attracted global attention, because of its palatability, high digestibility, nutritional value, and production benefits for herbivores in most countries (4, 5).

Ensiling, a simple and low-cost technique, has been traditionally and widely used for better nutrient preservation of fresh forages for year-round forage supply, particularly under unfavorable climatic conditions (6). The investigation by Wang et al. (7) revealed that ensiling fermentation may happen spontaneously as a result of microorganisms attached to the surface of the plants. In addition, adding microbial inoculants can speed up the fermentation progress, thus resulting in better silage quality and lower DM and nutrient losses during ensiling and the feed-out period. Commercial microbial inoculants are in common use in silage production, in which lactic acid bacteria (LAB) are the most promising microbial inoculants (8). In general, LAB inoculants increase the speed of fermentation and minimize nutrition losses during the initial phase of ensiling and prolong the ensiling time of ensilage in well-sealed silos (9). To date, oat silage studies on specific LAB inoculants and microbial community have been performed systematically (7, 10). Oliveira et al. (11) reported that only 5.7% of studies compared the effects of various types of *Lactobacillus* on oat silage quality, and 67% of studies tested the effect of Lactobacillus plantarum. In recent years, however, individual inoculants such as Lactiplantibacillus plantarum, Lentilactobacillus buchneri, Lacticaseibacillus paracasei, or Pediococcus acidilactici have been tested for oat silage fermentation and nutritional quality (7, 12, 13). Clearly, specific LAB inoculants have their unique functions. For instance, as a homofermentative LAB, Pediococcus acidilactici occupies a dominant position during the initial period of fermentation, resulting in the production of lactic acid to inhibit spoilage organisms (14), and as a functional genus of LAB in silage, *Lacticaseibacillus* can also directly restrain the growth of undesirable microorganisms (15). In addition, *Lactiplantibacillus*, one of the most commonly used inoculants, can rapidly produce lactic acid and lower the pH in forage fermentation, and *Lentilactobacillus* can enhance the aerobic stability of various forages (16). In the ensiling process, particularly in the initial phase of ensiling, the metabolisms of plant cells and microbes contribute to gas production (carbon dioxide [CO_2_] greater than 60%), resulting in greenhouse gas emissions and losses of DM and energy from the ensiled material (17). Chen et al. (18) found that Lactobacillus plantarum can decrease CO_2_ production by the control microbial community during silage fermentation.

Recently, detailed results regarding specific individual microbial inoculants have provided sufficient information to develop compound LAB in a more precise way (19). Therefore, studies on the performance of different LAB species combinations in terms of microbial community dynamics, silage fermentation characterization, feed value, and aerobic stability in the silo and feed-out periods are required.

Compound LAB inoculants have shown beneficial effects on silage quality for different whole crops and forage biomass silages (19). Several studies have shown that proper compound LAB inoculants can improve silage fermentation and nutritional quality (20). In fact, numerous commercial inoculants use combinations of LAB, which usually contain *Lentilactobacillus*, *Lactiplantibacillus*, *Pediococcus*, *Lacticaseibacillus*, etc., ensuring rapid fermentation and increasing silage stability in the silo and feed-out periods. However, studies on the application of different commercial inoculants on fermentation and nutritional quality of oat silage are scarce. Therefore, the community synergy of LAB and cleaner fermentation of oat silage prepared with microbial inoculant were studied.

## RESULTS

### Changes in chemical and microbial compositions of oat during ensiling.

Data on the nutrient composition, microbial community, and diversity index of pre-ensiled oat are summarized in Table 1, and data on the changes in chemical and microbial composition during ensiling (0 to 90 days), as affected by the LAB inoculants, are shown in Table 2. The DM content was not affected (*P > *0.05) by the inoculants, the ensiling duration, or their interaction. DM loss increased significantly (*P < *0.05) with ensiling duration. Higher DM loss was detected in LI2-treated silage on average in comparison with the control silage, whereas DM loss in LI1- and LI2-treated silages had no significant difference. The inoculant, ensiling duration, and their interaction altered (*P < *0.05) lactate buffer capacity (LBC), water-soluble-carbohydrate (WSC) content, and LAB and aerobic-bacterium counts in silages. The LBC of fresh oat was 250.84 meq/kg DM. Overall, a lower level (*P < *0.05) of LBC was observed for LI2-inoculated silage than for LI1- and control-treated silages. The level of LBC markedly varied during the ensiling process. The LI1-treated silage had higher (*P < *0.05) contents of WSC, while the control and LI2-inoculated silages showed no difference in WSC contents. The WSC content was significantly (*P < *0.05) influenced by the ensiling duration. However, at 1, 3, 6, and 90 days of fermentation, the WSC content of LI1-inoculated silage was significantly (*P < *0.05) greater than that of the control and LI2 silages. The inoculant and ensiling duration did not alter the content of crude protein (CP) in silages. However, the contents of neutral detergent fiber (NDF) and acid detergent fiber (ADF) altered due to ensiling duration. In particular, the NDF content in the LI1-inoculated silage significantly increased at 90 days compared to 1, 3, 6, and 15 days of ensiling. The numbers of yeast and aerobic bacteria were lower (*P < *0.05) in LI1-inoculated silage than control and LI2-inoculated silage. Moreover, the numbers of yeast and aerobic bacteria decreased (*P < *0.05) with increase in ensiling duration. Coliform bacteria in fresh oats exceeded 7 log_10_ CFU/g FM, declined to <5 log_10_ CFU/g FM in inoculated silage on the 1st day of ensiling, and disappeared after the 3rd day of fermentation, while coliform bacteria of control silage were detected continuously on the 3rd day of fermentation.

**TABLE 1:** Chemical composition, microbial community, and diversity index of fresh oat^*a*^

Parameter	Value
Dry matter (g/kg)	300.90 ± 5.46
Lactate buffering capacity (meq/kg DM)	250.84 ± 5.04
Water-soluble carbohydrates (g/kg DM)	73.06 ± 2.18
Crude protein (g/kg DM)	96.85 ± 4.67
Neutral detergent fiber (g/kg DM)	364.17 ± 63.49
Acid detergent fiber (g/kg DM)	179.00 ± 6.78
pH	6.20 ± 0.04
Lactic acid bacteria (log_10_ CFU/g FM)	5.92 ± 0.05
Aerobic bacteria (log_10_ CFU/g FM)	7.63 ± 0.10
Yeasts (log_10_ CFU/g FM)	6.35 ± 0.35
Coliform bacteria (log_10_ CFU/g FM)	7.10 ± 0.14
OTUs	228.25 ± 55.52
Shannon index	1.46 ± 0.40
Simpson index	0.46 ± 0.15
Chao1 index	340.59 ± 97.93
Coverage	0.9972 ± 0.0011

a The values are means and standard deviations for four replicates. FM, fresh matter; DM, dry matter; OTUs, operational taxonomic units.

**TABLE 2:** Chemical composition and microbial population of oat silage^*a*^

Item	Treatment	Value on ensiling day						Mean	SEM	*P* value		
		1	3	6	15	35	90			I	D	I × D
DM (g/kg)	Control	306.58A	294.99bB	298.88AB	300.49AB	299.97AB	292.49B	298.90b	4.14	0.0570	0.0783	0.1098
	LI1	305.79AB	300.25abAB	304.74AB	315.06A	293.20B	304.94AB	304.00a				
	LI2	300.24AB	304.23aA	301.10AB	298.07AB	298.41AB	291.44B	298.92b				
	Mean	304.21A	299.82AB	301.57AB	304.54A	297.20AB	296.29B					

DM loss (g/kg DM)	Control	4.51F	8.03aE	13.20D	21.20C	60.49B	86.90A	32.39b	1.92	0.0703	<0.0001	0.2545
	LI1	4.26E	7.59abDE	13.30D	23.01C	63.04B	96.22A	34.57ab				
	LI2	4.81E	7.10bE	13.24D	22.98C	63.77B	96.49A	34.73a				
	Mean	4.53E	7.57E	13.25D	22.40C	62.43B	93.20A					

Lactate buffering capacity (meq/kg DM)	Control	298.66bA	217.84aD	227.14aD	238.25aC	217.72aD	257.51bB	242.85a	3.22	<0.0001	<0.0001	<0.0001
	LI1	326.86aA	208.44bD	207.17cD	220.77bC	198.37bD	277.43aB	239.84a				
	LI2	193.49cE	201.58bD	218.64bB	218.94bB	209.68abC	257.68bA	216.67b				
	Mean	273.00A	209.29E	217.65D	225.99C	208.59E	264.21B					

Water-soluble carbohydrate (g/kg DM)	Control	61.65bAB	66.52bA	58.08bB	55.07B	35.21bC	41.19bC	52.95b	2.43	<0.0001	<0.0001	0.0074
	LI1	73.83aA	74.23aA	71.50aAB	63.10B	43.31aC	64.44aB	65.07a				
	LI2	66.30bA	62.04bA	60.73bA	52.69B	44.08aC	46.48bBC	55.39b				
	Mean	67.26A	67.60A	63.44A	56.96B	40.87D	50.70C					

Crude protein (g/kg DM)	Control	100.60	95.94b	98.69	100.29	101.99a	100.83	99.72	1.74	0.4369	0.9562	0.0044
	LI1	98.96	98.29b	99.47	98.18	100.68a	99.69	99.21				
	LI2	97.10BC	105.37aA	97.37BC	99.30B	95.17bC	96.27BC	98.43				
	Mean	98.89	99.87	98.51	99.26	99.28	98.93					

Neutral detergent fiber (g/kg DM)	Control	267.71AB	277.43A	273.65A	240.47bB	264.72AB	285.50A	268.25b	17.62	0.0085	0.0014	0.1132
	LI1	283.78BC	261.06C	271.90C	256.54abC	348.45AB	358.90A	296.77a				
	LI2	290.19	290.60	283.79	288.05a	319.59	307.65	296.64a				
	Mean	280.56B	276.36B	276.45B	261.69B	310.92A	317.35A					

Acid detergent fiber (g/kg DM)	Control	149.78AB	159.16A	154.48A	133.06bB	151.46bAB	166.71A	152.44	7.60	0.1352	0.0287	0.4508
	LI1	158.55AB	146.22B	153.54AB	148.28abB	176.32aA	163.20AB	157.68				
	LI2	157.87	164.17	161.56	154.27a	161.86ab	168.34	161.35				
	Mean	155.40AB	156.52AB	156.53AB	145.20B	163.21A	166.08A					

Lactic acid bacteria (log_10_ CFU/g FM)	Control	9.21bAB	9.24aA	8.71abC	8.96aABC	8.87aBC	7.91aD	8.82a	0.09	<0.0001	<0.0001	0.0017
	LI1	9.38aA	8.98bB	8.40bC	8.47bC	8.45bC	7.32bD	8.50b				
	LI2	9.40aA	9.31aA	9.01aB	8.87aB	9.03aB	7.72aC	8.89a				
	Mean	9.33A	9.18B	8.71C	8.77C	8.78C	7.65D					

Aerobic bacteria (log_10_ CFU/g FM)	Control	9.36A	9.1175aB	8.86C	8.34aD	8.38aD	7.73aE	8.63a	0.07	<0.0001	<0.0001	<0.0001
	LI1	9.39A	8.81bB	8.71B	7.66cC	7.83bC	7.31bD	8.28b				
	LI2	9.41A	9.27aA	8.77B	7.95bD	8.41aC	7.79aD	8.60a				
	Mean	9.39A	9.07B	8.78C	7.98E	8.20D	7.61F					

Yeasts (log_10_ CFU/g FM)	Control	9.33A	9.24aAB	9.01B	8.50abC	9.03B	7.64D	8.79a	0.11	0.0003	<0.0001	0.1017
	LI1	9.21A	8.83bA	8.86A	8.17bB	9.08A	7.29C	8.57b				
	LI2	9.39A	9.37aA	8.98BC	8.71aC	9.13AB	7.34D	8.82a				
	Mean	9.31A	9.15AB	8.95C	8.46D	9.08BC	7.42E					

Coliform bacteria (log_10_ CFU/g FM)	Control	7.87	3.87	N	N	N	N		0.12			
	LI1	4.83	N	N	N	N	N					
	LI2	4.43	N	N	N	N	N					
	Mean	5.71	N	N	N	N	N					

a DM, dry matter; FM, fresh matter; I, inoculant; D, ensiling days; I × D, interaction between additive and ensiling days; N, not detected. Means with different letters in the same row (A to F) or column (a to c) differ significantly (*P *< 0.05).

### Fermentation characteristics of oat silages during ensiling.

The inoculants, ensiling duration, and their interaction significantly (*P < *0.05) influenced pH and acetic acid, ammonia N, and gas production of oat silages (Table 3). The LI1-inoculated silages had lower (*P < *0.05) pH than control and LI2-inoculated silages in this investigation. The pH values in control and LI2-inoculated silages gradually decreased, reaching their lowest levels at 15 days of ensiling, while they significantly increased from 15 to 90 days. In contrast, the pH of LI1 silages rapidly decreased to below 4.0 at 3 days of ensiling and remained at their lowest level (<4.0) until 15 days. Moreover, the pH values of LI1 silages at 35 and 90 days were lower (*P < *0.05) than those of LI2 silages. Ensiling duration and interaction of ensiling duration and inoculant significantly (*P < *0.05) affected lactic acid concentration. Unexpectedly, lactic acid and acetic acid contents did not vary between LI1- and LI2-inoculated silages. However, the lactic acid and acetic acid contents were significantly (*P < *0.05) higher in the inoculated silages than in the control. The lactic acid content in LI1-inoculated silages remained high (>51 g/kg DM) at 1 to 6 days of fermentation and then significantly (*P < *0.05) declined, reaching its lowest level at 90 days of ensiling. Consistently, on 1 and 3 days of ensiling, lactic acid content in the LI2 treatment was significantly (*P < *0.05) lower than that in LI1 treatment; nevertheless, lactic acid content between LI1and LI2 treatments had no significant difference (*P > *0.05) on 6 and 15 days of ensiling. The acetic acid contents in all treatments consistently increased during ensiling. Propionic acid content ranging from 2 to 9 g/kg DM was detected in LI1-inoculated silage at 35 and 90 days of ensiling. Inoculations of the LAB decreased the ammonia N concentrations of oat silages. On average, the ammonia N concentration gradually increased during ensiling. At 1, 3, 15, and 35 days of ensiling, the LI1- and LI2-inoculated silages had significantly lower (*P < *0.05) ammonia N concentrations than the control. However, at 6 and 90 days of ensiling, the LI1-inoculated silage had significantly lower (*P < *0.05) ammonia N concentrations than control and LI2-inoculated silages. The gas production was lower (*P < *0.05) in LI1-inoculated silage than in control and LI2-inoculated silages. The gas production of each treatment increased rapidly in the initial phase of fermentation but gradually decreased with the prolongation of fermentation.

**TABLE 3:** Fermentation characteristics of oat silage^*a*^

Item	Treatment	Value on ensiling day						Means	SEM	*P* value		
		1	3	6	15	35	90			I	D	I × D
pH	Control	4.61aB	4.15aC	4.09aD	4.12aCD	4.69bA	4.68bA	4.39a	0.01	<0.0001	<0.0001	<0.0001
	LI1	4.11bC	3.98bD	3.98cD	3.97bD	4.48cB	4.68bA	4.20c				
	LI2	4.10bC	4.09aCD	4.05bD	4.12aC	4.78aA	4.72aB	4.31b				
	Mean	4.27C	4.07D	4.04E	4.07D	4.65B	4.69A					

Lactic acid (g/kg DM)	Control	33.12cBC	36.50cB	46.72bA	32.43C	18.80bD	15.96aD	30.59b	0.81	0.1925	<0.0001	<0.0001
	LI1	51.28aB	57.25aA	57.72aA	31.27C	23.05aD	7.66bE	38.04a				
	LI2	46.17bC	52.39bB	57.59aA	32.71D	20.08bE	15.63 aF	37.43a				
	Mean	43.52C	48.71B	54.01A	32.13D	20.64E	13.08F					

Acetic acid (g/kg DM)	Control	5.42bD	6.54bC	7.12bC	11.86cB	26.76aA	25.92bA	13.93b	0.41	<0.0001	<0.0001	<0.0001
	LI1	5.55bE	7.78abD	8.35aD	17.22aC	25.11bB	30.29aA	15.72a				
	LI2	10.19aD	8.07aE	7.87abE	16.08bC	28.05aA	24.91bB	15.86a				
	Mean	7.05D	7.46CD	7.78C	15.05B	26.64A	27.04A					

Propionic acid (g/kg DM)	Control	N	N	N	N	N	N	N				
	LI1	N	N	N	N	2.13	8.51					
	LI2	N	N	N	N	N	N	N				
	Mean	N	N	N	N	N	N	N				

Ammonia nitrogen (g/kg TN)	Control	19.19aD	23.99aBC	22.88aC	27.49aB	37.54aA	36.20aA	27.88a	0.89	<0.0001	<0.0001	0.0340
	LI1	15.82bD	17.85bCD	19.91bBC	21.44bB	32.72bA	32.09bA	23.30c				
	LI2	15.64bE	20.53bD	24.65aB	22.60bC	33.80bA	34.55abA	25.29b				
	Mean	16.88D	20.79C	22.48B	23.84B	34.69A	34.28A					

Gas production (mL)	Control	281.25aA	307.50aA	292.50aA	190.00aB	85.00C	55.00bD	201.88a	9.72	<0.0001	<0.0001	<0.0001
	LI1	237.50bB	275.00bA	182.50bC	90.00bD	50.00E	75.00aD	151.67c				
	LI2	262.50aA	265.00bA	207.50bB	130.00bC	67.50D	45.00bD	162.92b				
	Mean	260.42B	282.50A	282.50C	136.667D	136.67E	58.33E					

a DM, dry matter; TN, total nitrogen; I, inoculant; D, ensiling days; I × D, the interaction between additive and ensiling days; N, not detected. Means with different letters in the same row (A to F) or column (a to c) differ significantly (*P *< 0.05).

### Bacterial community diversity, compositions, and successions in oat silages during ensiling.

Tables 1 and 4 show richness and diversity indicators of bacterial communities in fresh oat and silage. The ensiling duration, inoculant, and their interaction significantly (*P < *0.05) influenced numbers of operational taxonomic units (OTUs) and Chao1, Shannon, and Simpson indexes of silages. In general, lower numbers of OTUs and lower Chao1 and Shannon indexes were detected in the LI2 silages than control and LI1 silages. The average numbers of OTUs and Chao1 indexes gradually decreased until the 35th day but increased significantly after 90 days of ensiling. Higher Shannon indexes were observed in LI1 silages than in control and LI2 silages at 15 days of fermentation. After 90 days of ensiling, control silages had the highest Shannon index compared to LAB-inoculated silages. At 6 and 15 days of fermentation, lower Simpson indexes were observed in the LI1 silages. High Good’s coverage (>99.70%) was detected in all samples.

**TABLE 4:** Richness and diversity indexes of microbial community in oat silage^*a*^

Index	Treatment	Value on ensiling day						Mean	SEM	*P* value		
		1	3	6	15	35	90			I	D	I × D
OTUs	Control	307.50AB	344.75aA	259.50abC	292.75aBC	192.00D	275.50aBC	278.67a	12.47	<0.0001	<0.0001	0.0025
	LI1	331.50A	301.75abAB	296.00aAB	280.50aBC	180.50D	239.25aC	271.58a				
	LI2	324.25A	271.50bB	237.50bC	248.25bBC	164.00D	181.00bD	237.75b				
	Means	321.08A	306.00A	264.33B	273.83B	178.83D	231.92C					
Shannon	Control	1.90B	2.18aA	1.91aB	1.95bB	1.46C	1.53aC	1.82a	0.07	<0.0001	<0.0001	<0.0001
	LI1	1.95A	1.66bB	2.16aA	2.17aA	1.53B	1.30bC	1.80a				
	LI2	2.02A	1.63bB	0.90bD	1.72cB	1.39C	1.28bC	1.49b				
	Means	1.96A	1.82B	1.65C	1.95A	1.46D	1.37D					
Simpson	Control	0.30ABC	0.22bC	0.28bBC	0.26aC	0.37abA	0.36AB	0.30b	0.03	<0.0001	<0.0001	<0.0001
	LI1	0.25C	0.37aB	0.18cC	0.17bC	0.34bB	0.46A	0.29b				
	LI2	0.27D	0.36aC	0.68aA	0.28aD	0.42aBC	0.45B	0.41a				
	Means	0.27D	0.32C	0.38B	0.24D	0.37B	0.42A					
Chao1	Control	450.14AB	521.13aA	430.53abB	455.39aAB	328.58C	433.04aB	436.47a	22.36	<0.0001	<0.0001	0.0026
	LI1	485.81A	436.22bAB	447.70aAB	433.63aAB	319.57C	400.38aB	420.55a				
	LI2	511.68A	433.58bB	377.65bB	400.58bB	261.11C	275.62bC	376.70b				
	Means	482.54A	463.64AB	418.63C	429.87BC	303.09E	369.68D					
Coverage	Control	0.9981AB	0.9978bAB	0.9982bAB	0.9970B	0.9987A	0.9983bAB	0.9980b	0.0003	0.0376	0.0013	0.2682
	LI1	0.9979C	0.9981aBC	0.9980bC	0.9983BC	0.9987A	0.9985bAB	0.9983ab				
	LI2	0.9978E	0.9982aD	0.9986aBC	0.9983CD	0.9990A	0.9989aAB	0.9985a				
	Means	0.9980C	0.9980C	0.9983BC	0.9979C	0.9988A	0.9985AB					

a OTUs, operational taxonomic units; I, inoculant; D, ensiling days; I × D, the interaction between additive and ensiling days. Means with different letters in the same row (A to D) or column (a to c) differ significantly (*P *< 0.05).

**FIG 1:**
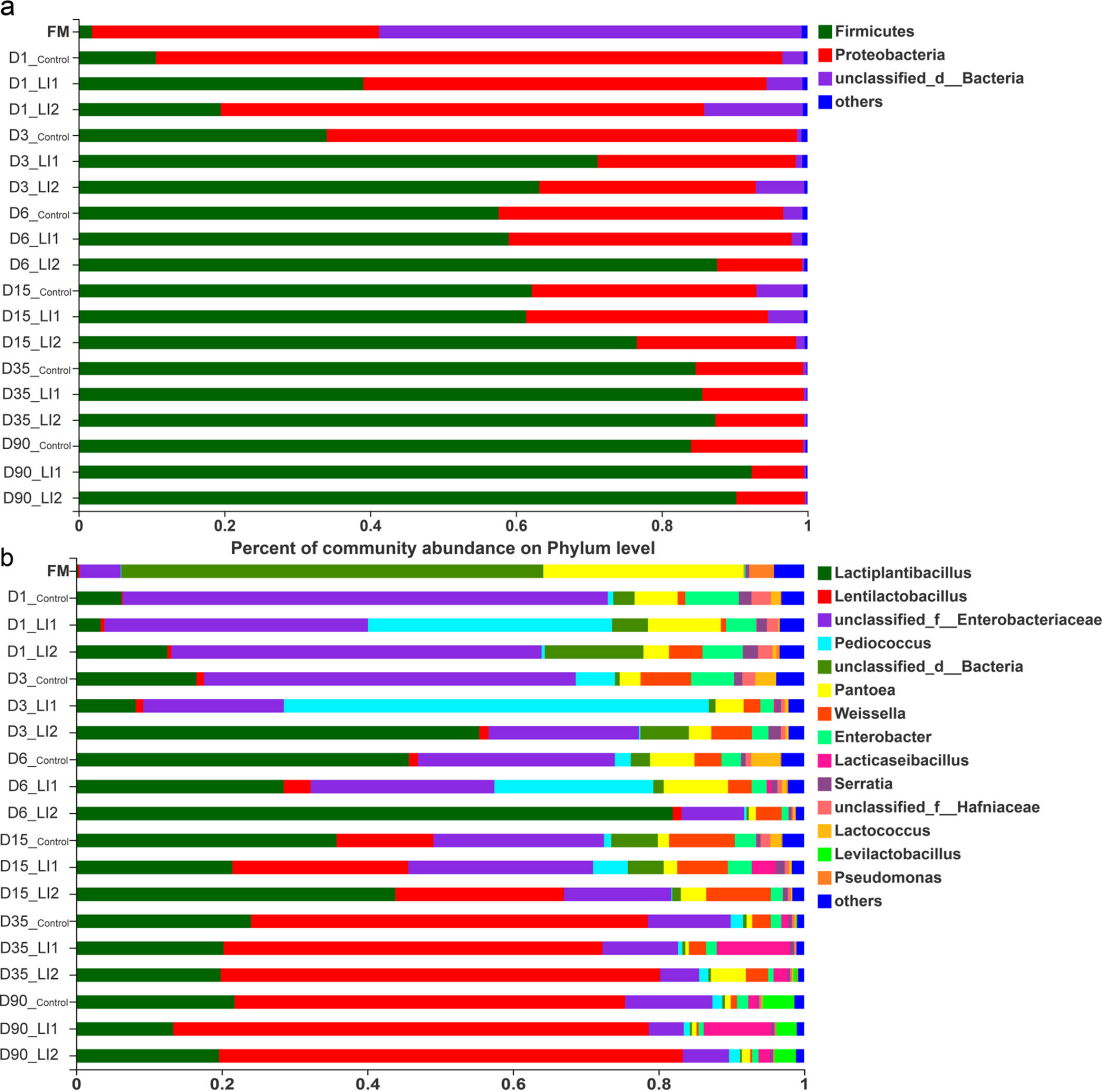
Relative abundance of bacteria community in oat and silage at the phylum (a) and genus (b) levels across different groups and fermentation times. LI1, silage inoculated with *Lactiplantibacillus plantarum*, *Lacticaseibacillus casei*, Lentilactobacillus buchneri, and Pediococcus acidilactici; LI2, silage inoculated with *Lactiplantibacillus plantarum* and Lentilactobacillus buchneri; D1, D3, D6, D15, D35, and D90, days 1, 3, 6, 15, 35, and 90 of silage, respectively.

Figure 1a shows the bacterial community composition at the phylum level in oat silage. Notably, *Proteobacteria* and unclassified bacteria were both dominant in the FM group, whereas *Proteobacteria* and *Firmicutes* played a predominant role in silages. The relative abundance of *Firmicutes* increased and *Proteobacteria* declined during the ensiling process. Moreover, the LI1 inoculant decreased the relative abundance of *Proteobacteria* and increased that of *Firmicutes* after 1 and 3 days of ensiling. However, the LI2-inoculated silage showed a low relative abundance of *Proteobacteria* and a high relative abundance of *Firmicutes* at 6 and 15 days of ensiling compared to control and LI1 silages. At the later stages of ensiling (35 and 90 days), the relative abundances of *Firmicutes* in LI1 and LI2 treatments reached more than 85%, and there was no significant difference between them.

**FIG 2:**
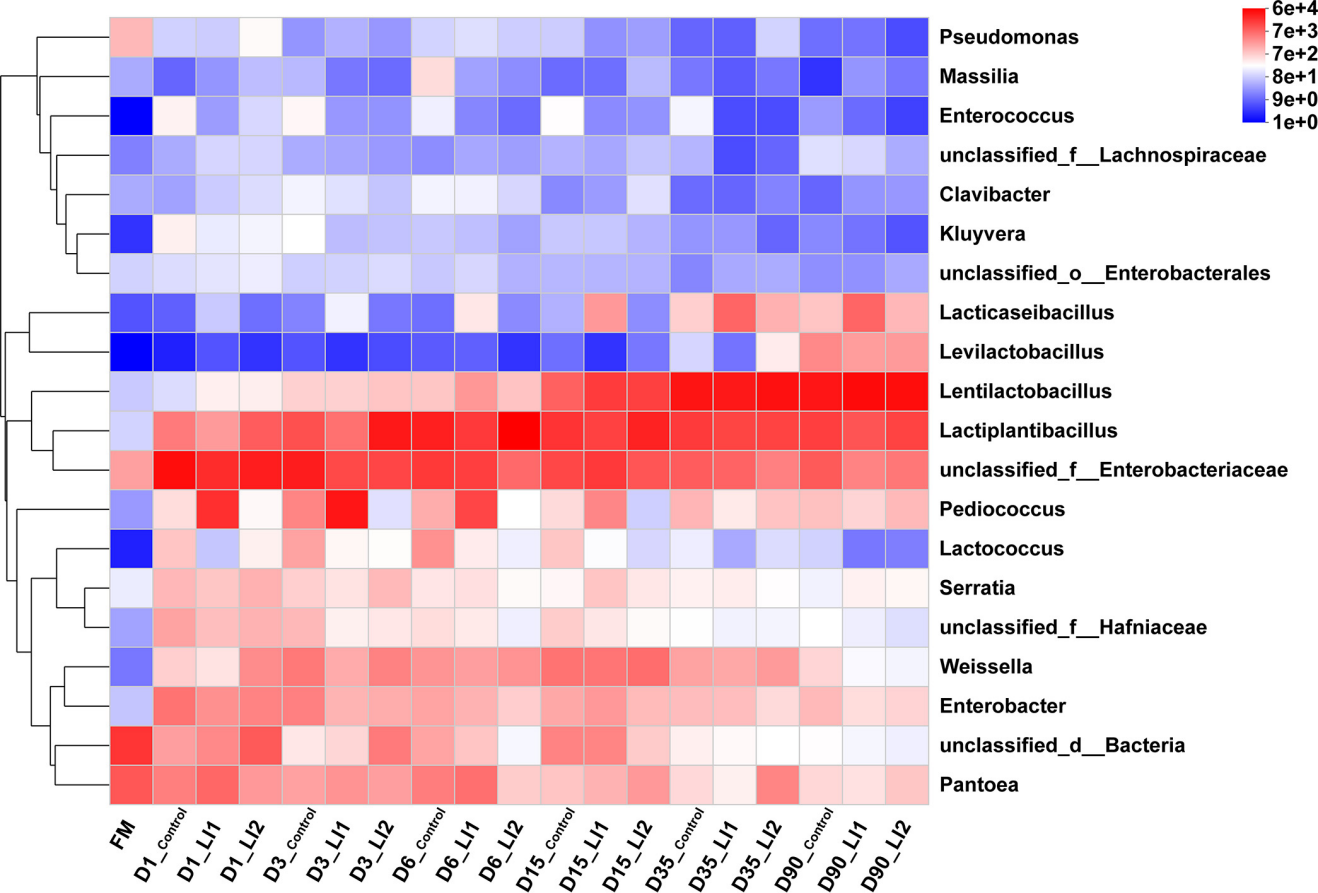
Heat map of prominent bacterial genera (20 most abundant genera) for oat silage before and after ensiling for 1, 3, 6, 15, 35, and 90 days with or without LI1 and LI2.

**FIG 3:**
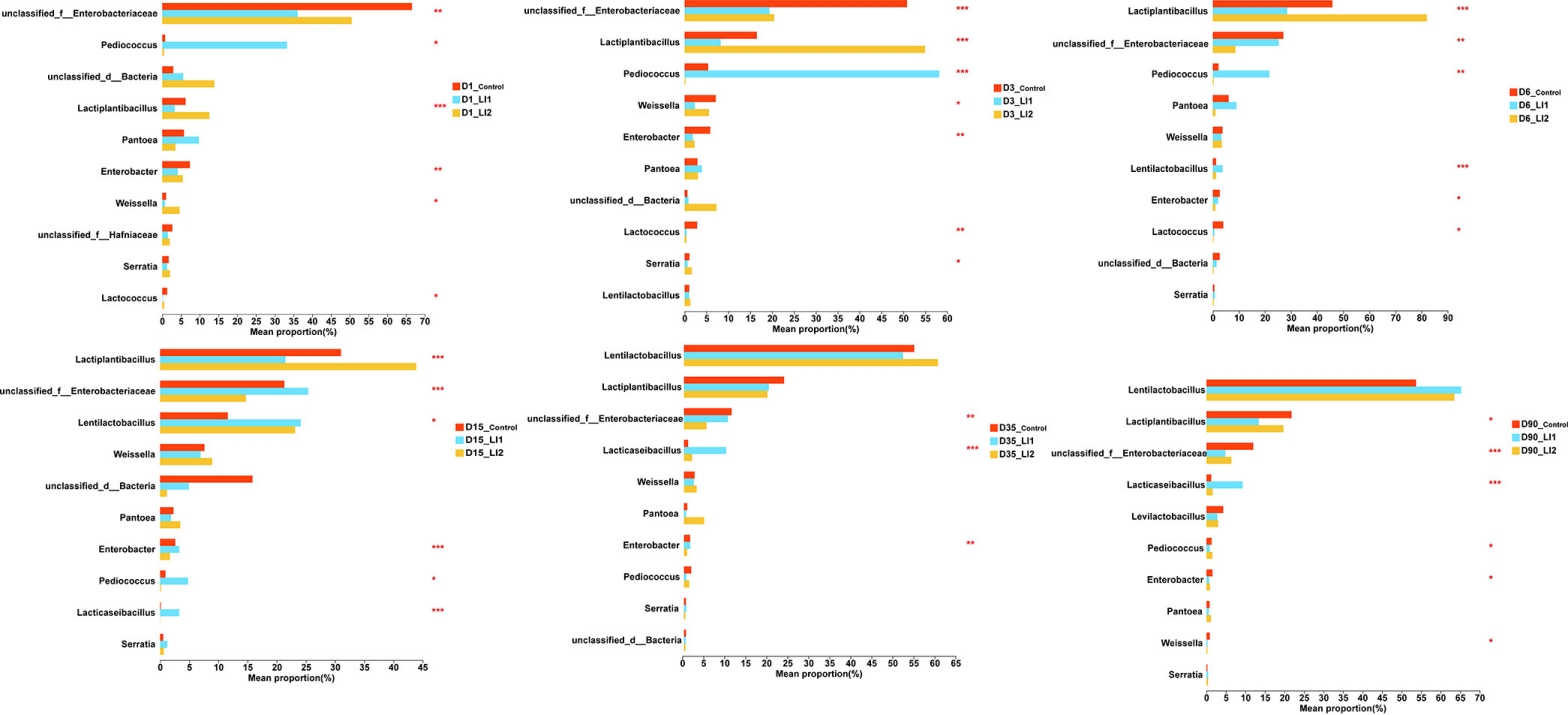
One-way analysis of variance bar plots of the 10 most abundant genera among different oat silage treatments. *, 0.01 < *P* ≤ 0.05; **, 0.001 < *P* ≤ 0.01; ***, *P* ≤ 0.001.

Figures 1b, 2, and 3 display intuitional and statistical comparison of the bacterial compositions at genus level across different treatments and ensiling days. The unclassified bacteria and *Pantoea* dominated the fresh oat, with a relative abundance of more than 57% and 27%, respectively. The relative abundance of *Lactobacillus* in the fresh oat was less than 1%. Control silage had a higher (*P < *0.05) percentage of members of the family *Enterobacteriaceae* than the LAB-inoculated silages at 1 and 3 days of ensiling. *Enterobacteriaceae* were gradually replaced by *Lactiplantibacillus* and *Lentilactobacillus* in the control group after 6 days of ensiling. *Lentilactobacillus* and *Lactiplantibacillus* play a predominant role at 90 days of ensiling in control group. Relative abundances of *Enterobacteriaceae* also decreased during ensiling in inoculated silages. The relative abundance of *Enterobacteriaceae* in control treatment at 1, 3, and 90 days of ensiling was significantly (*P < *0.01) greater than that in the LAB-inoculated treatments (Fig. 3). Nevertheless, the relative abundance of *Enterobacteriaceae* in the LI1 treatment was the same as in the control but higher (*P < *0.01) than in the LI2 treatment at 6, 15, and 35 days of ensiling. At the 1st day of fermentation, *Enterobacteriaceae* and *Pediococcus* were dominant in the LI1 group, while only *Pediococcus* dominated at 3 days of fermentation and inhibited the growth of *Enterobacteriaceae*. Much more interestingly, *Lactiplantibacillus* was just starting to become the predominant genus, followed by *Enterobacteriaceae* and *Pediococcus*, at 6 days of ensiling in the LI1 group, and during the end period of ensiling, *Lentilactobacillus* was the most predominant, followed by *Lactiplantibacillus*. *Lacticaseibacillus* showed significantly higher (*P < *0.001) relative abundance at only the late stage of ensiling (15, 35, and 90 days) in the LI1 group than in the LI2 and control groups. Interestingly, for the LI2 group, the silage was dominated by *Enterobacteriaceae* at 1 day of ensiling. However, *Lactiplantibacillus* became the most predominant in LI2 at 3, 6, and 15 days of ensiling and then was replaced by *Lentilactobacillus* at 35 and 90 days of ensiling.

### Relationship between silage bacterial taxonomic profile and quality variables.

**FIG 4:**
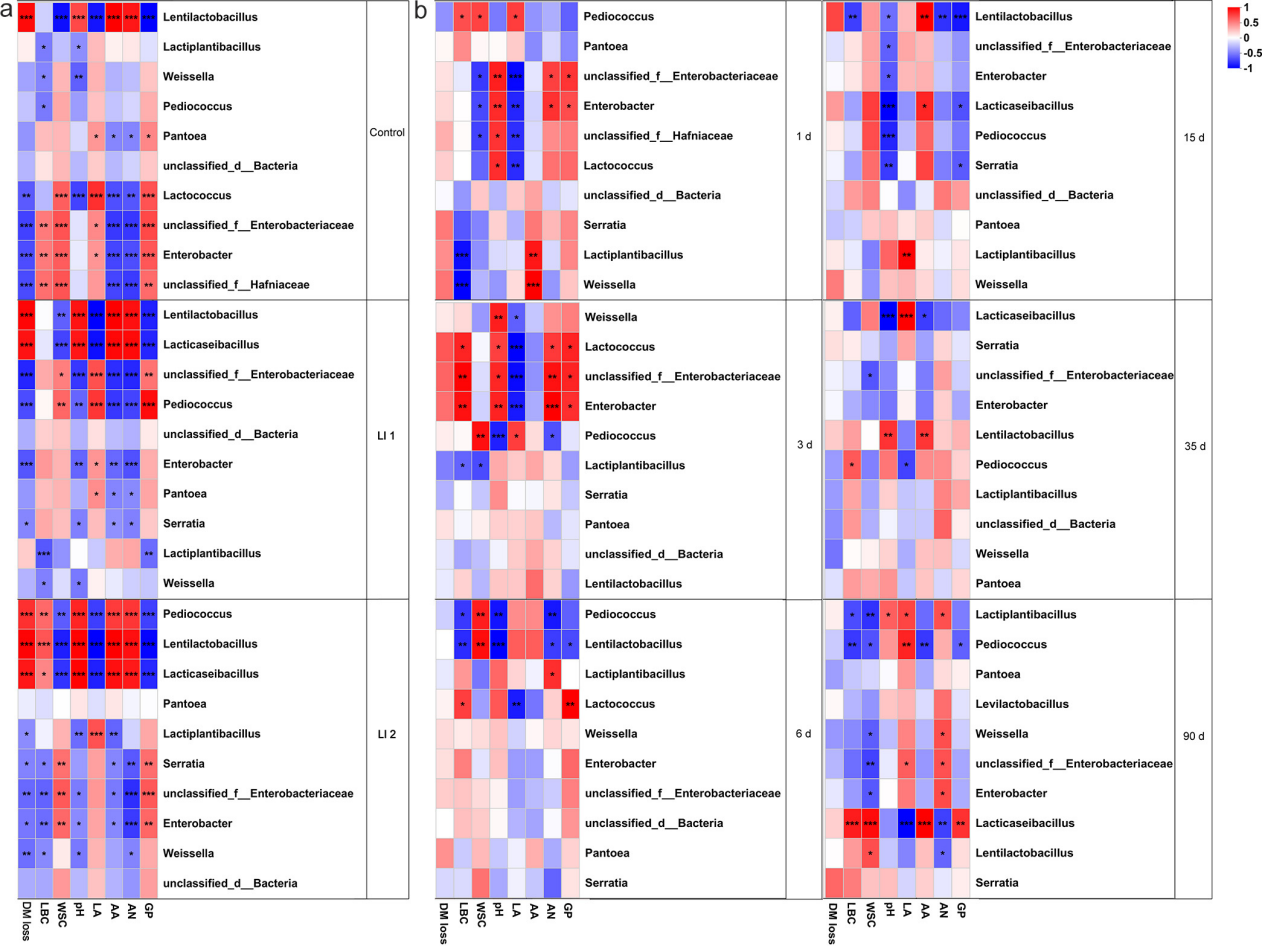
Association analysis between bacterial genera and quality variables. Quality variables are displayed horizontally, and the bacterial genera are displayed vertically. The corresponding value of the middle heat map is the Pearson correlation coefficient *r*, which ranges between −1 and 1; an *r* value of <0 indicates a negative correlation (blue), and a value of >0 indicates a positive correlation (red). *, *P* < 0.05; **, *P* < 0.01; ***, *P* < 0.001. (a) Correlations between bacterial genera and quality variables in different silage groups. (b) Correlations between bacterial genera and quality variables at different ensiling time points (1, 3, 6, 15, 35 and 90 days of ensiling). LBC, lactate buffer capacity; WSC, water-soluble carbohydrate; LA, lactic acid; AA, acetic acid; AN, ammonia nitrogen; GP, gas production.

Figure 4a shows the results of association analysis between the major quality variables and the top10 bacterial genera. Among the three treatments, *Enterobacteriaceae* had a positive correlation with gas production, and Enterobacter had negative correlation with DM loss; *Lentilactobacillus* had a positive correlation (*P < *0.05) with pH value, acetic acid, ammonia N, and DM loss and a negative correlation (*P < *0.05) with lactic acid, WSC, and gas production in all groups. This result was the same as that for *Lacticaseibacillus* from LI1 and LI2 treatments and *Pediococcus* only from the LI2 treatment and the opposite of that for *Pediococcus* from the LI1 treatment. *Lactiplantibacillus* was negatively correlated with LBC and gas production of the LI1 treatment and pH and acetic acid of the LI2 treatment. In addition, Fig. 4b shows the effect of the top 10 bacterial genera on the main quality variables at different ensiling days. The correlations between bacterial genera and quality variables were more complex when the silage fermentation started (1 and 3 days) and finished (35 and 90 days) and simpler in the intermediate stage (6 and 15 days) of ensiling.

### Predicted functions of bacterial communities during oat ensiling.

**FIG 5:**
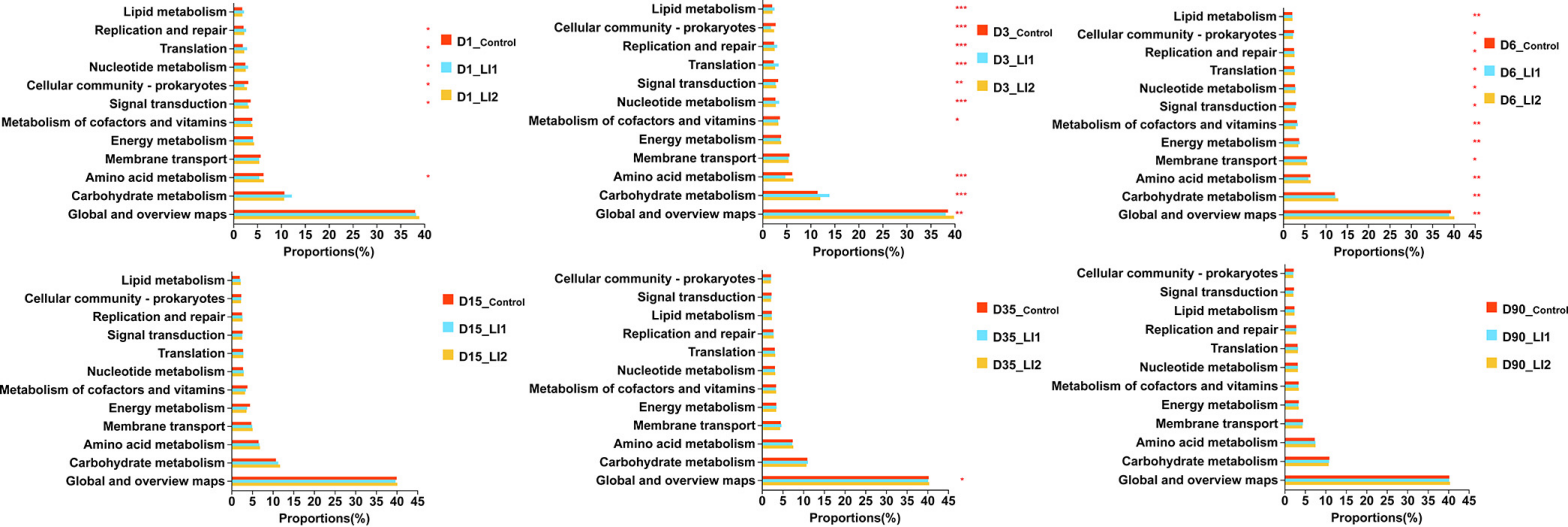
Level 2 KEGG orthologous sequences of ensiled oat as influenced by additives and ensiling time. *, 0.01 < *P* ≤ 0.05; **, 0.001 < *P* ≤ 0.01; ***, *P* ≤ 0.001. Functional prediction of bacterial changes in oat after fermentation was made using PICRUSt2.

Figure 5 displays the bacterial metabolic functions determined with PICRUSt2 and based on KEGG (Kyoto Encyclopedia of Genes and Genomes) pathways. We obtained 46 metabolic categories, which belong to the metabolism pathway, environmental information processing pathway, genetic information processing pathway, and cellular process pathway, among the three treatments. Figure 5 shows the differences of the first 12 metabolic functions with high abundance in three kinds of silages after 1, 3, 6, 15, 35, and 90 days of ensiling. The global and overview maps pathway showed the most significant role in metabolic categories, followed by carbohydrate and amino acid metabolic pathways in all silages after ensiling. The global and overview maps predominated (*P < *0.05) in the LI2-inoculated silage at 3, 6, and 35 days of ensiling. In the LI1 treatment, the amino acid metabolism was significantly lower (*P < *0.05) than that in control and LI2 treatments at 1, 3, and 6 days of ensiling. The carbohydrate metabolism pathway was more (*P < *0.05) dominant in the LI1-treated silages at 3 days than in LI2 silages, while the opposite result was found for LI1 at 6 days of ensiling. In general, at the early stage of ensiling (before 6 days), there were at least 6 metabolic pathways with significant differences at each ensiling day, but there were few metabolic pathways with significant differences in the late period of ensiling.

## DISCUSSION

The DM content at ensiling is a crucial index for fermentation characteristics and preservation of nutritional value of forages (21). The oat was ensiled at a high DM content (301 g/kg), and there were few changes in DM content due to ensiling duration and LAB inoculants. As reported for barley silages, there are few changes in DM content in a well-preserved silage process (22). The higher DM content of LI1-inoculated silage, compared with the control and LI2-treated silages in our study could be related to the effectiveness of LAB inoculation, which restricts extensive fermentation (11). Producing preferred-quality silage while minimizing DM loss is a challenge (19). In general, the DM loss ranging from 2% to 6% caused by lactic acid fermentation is a bit low (23). In our study, the DM losses increased from 0.4% to 6% during 1 to 35 days of ensiling in all treatments, showing a restricted fermentation, which might be due to the predominant lactic acid fermentation pathway in the silages. Unusually, the average DM loss of oat silages in the three treatments was up to about 9% after 90 days of ensiling. This might be related to the higher DM content (≥300 g/kg) of the oat in the silage and to the relative abundance of Lentilactobacillus buchneri, resulting in higher fermentative losses (4). In addition, the higher levels of DM loss in LI2-treated silage compared to control silage might be attributed to the significantly positive correlation between DM loss and *Lentilactobacillus*, *Lacticaseibacillus*, and *Pediococcus* in LI2 treatment (Fig. 4). The WSC was used mainly by microorganisms for energy metabolism during the ensiling process (13). The content of WSC (73.06 g/kg) of fresh oat in the present study was enough for fermentation (24). Moreover, inoculation with LI1 supported efficient fermentation during ensiling, resulting in higher WSC levels in this silage than in control and LI2 silages. The lower WSC content in LI2 and control silages reflects more extensive fermentation of WSC than in LI1 silage, which might be related to the relative abundance of *Lentilactobacillus* (25). The decreases in WSC contents in all silage could be related to the conversion of WSC into organic acid by LAB during ensiling (26). The LAB-treated silages had higher ADF contents than the control silage. The reason for this is not entirely clear. It may be due to the addition of LAB, which consumes a large amount of fermentation substrates such as WSC, resulting in an increase in the relative content of ADF. The LI1-inoculated silage had higher NDF content at 90 days than at 1, 3, 6 and 15 days of ensiling. Generally, as silage fermentation progresses, it is difficult for microorganisms to decompose the fiber components, but they can decompose part of the soluble carbohydrates into carbon dioxide, water, ethanol, etc. (19), which can easily cause the loss of dry matter and thus lead to the relatively high content of fiber components in the silage.

Table 1 shows that the epiphytic LAB count in fresh oat exceeded the lowest requirement (5 log_10_ CFU/g FM) for preferred quality of silage preparations (27). However, the yeast count in fresh forage was above 6 log_10_ CFU/g FM, which increases the possibility of aerobic spoilage, dry matter loss, and CO_2_ production during ensiling. Cheng et al. (12) reported lower counts of LAB and yeasts in fresh oats grown in different regions than in this experiment. This may be related to the difference in growing conditions, such as differences in geographical location and ambient temperature.

The pH value of silage is a key indicator of silage quality, and the benchmark for high-quality silage fermentation is a pH below 4.2 (28). In the present study, a rapid acidification, with lower pH and higher lactic acid was observed in LAB-treated groups during initial stages of fermentation (before 15 days of ensiling), especially in the LI1 group. This was probably because the LI1 inoculant contains two homofermentative LAB (*Lactiplantibacillus plantarum* and Pediococcus acidilactici), which have been reported to speed up the fermentation during initial periods of fermentation by more efficiently shifting WSC into lactic acid, resulting in a rapid drop in pH (19). Conversely, silages with higher relative abundances of heterofermentative *Lentilactobacillus* have a high acetic acid content because of their ability to change some lactic acid into acetic acid (29). The acetic acid content increased progressively with the ensiling duration, which might be because of the obvious enhancement of the relative abundance of *Lentilactobacillus* at the late period of ensiling (15, 35, and 90 days). Chen et al. (30) suggested that a better alternative LAB inoculant for ensiling is mainly marked by fast increase, resistance to low pH, and rapid production of preferred metabolites. The present study showed that a rapid drop in pH and quick production of lactic acid in oat silage were promoted by LI1 inoculation. Ammonia N is an important index for reflecting the degree of crude protein degradation based on fermentation of *Clostridium* in the silage (31, 32). The LAB inoculant reduced the ammonia N content of silages, which indicated that LAB restricted the reproduction of undesirable microbes in silage, resulting in a decrease in the degradation of protein (33). As reflected by the negative correlation between ammonia N and *Pediococcus*, *Lacticaseibacillus*, and *Lentilactobacillus* during fermentation (Fig. 4). Overall, the ammonia N concentration gradually increased during ensiling until 35 days of ensiling, indicating that stable conditions were achieved after 35 days of ensiling, stopping further proteolysis.

Gas production (GP) during ensiling not only is bad for the environment but also causes dry matter loss in silage. A previous study found that approximately 6.0 L/kg FM of gas was produced after 60 days of ensiling (34) and mainly emitted at the early stages of fermentation (35). The present study indicated that the average gas production in all silages was greater during the initial stage (1 and 3 days) and then sharply declined at later ensiling days. Another study stated that the relative abundance of *Lactococcus* had a positive correlation with gas production (18). The higher gas production during the initial stage in our study was also associated with a higher relative abundance of *Lactococcus*. Moreover, the decrease of the relative abundance of *Enterobacteriaceae* and increase of the relative abundances of *Lentilactobacillus* and *Lacticaseibacillus* during ensiling were observed at all silages. Association analysis showed that the relative abundance of *Enterobacteriaceae* was positively correlated with GP, whereas the relative abundance of *Lentilactobacillus* and *Lacticaseibacillus* was negatively correlated with GP (Fig. 4). It could further explain the reduction in gas production at the later stage of fermentation.

All the results above verify that LI1 improved the fermentation quality of oat silage. Similar findings were showed by Li et al. (36) and Na et al. (33), who suggested that the inoculation of exogenous LAB (*Lacticaseibacillus paracasei*, Pediococcus acidilactici, Lentilactobacillus buchneri and *Lactiplantibacillus plantarum*) improved the fermentation quality of silages. Generally, silages with good fermentation quality have low alpha diversity (37). In the present study, the higher Shannon index in LI1-treated silage at 6 and 15 days of ensiling compared with that in LI2 silage was inconsistent with the lower pH and relatively high fermentation quality, which can be explained by the higher relative abundance of *Enterobacteriaceae* in LI1-inoculated silages. A previous study reported that a combination of homofermentative *Lactiplantibacillus plantarum* and heterofermentative Lentilactobacillus buchneri was advantageous to control the early fermentation stage by reducing the activity of enterobacteria and other aerobic bacteria (19). This result was confirmed in our bacterial community diversity and composition analysis data (Fig. 1 to 3). Romero et al. (38) reported that the application of inoculant enhanced oat silage quality partially by a change in the microbial community composition during ensiling. During the growing season, *Proteobacteria* are the main bacterial group in plant leaves, followed by *Firmicutes* in some cases (39). In the present study, the same phenomenon was found in fresh oat. In addition, Yuan et al. (40) suggested that lactic acid fermentation happens naturally in an anaerobic environment. Likewise, Eikmeyer et al. (41) and Ogunade et al. (42) also found that the epiphytic microorganisms ferment the available nutrients in forage, so as to achieve the production of organic acids, reduction of pH, and prevention of harmful microorganisms’ propagation. In this investigation, the relative abundance of *Lactobacillus* in the fresh oat was less than 1%, while the total relative abundance of *Lentilactobacillus* and *Lactiplantibacillus* in the control increased sharply to 46.97% at 6 days of ensiling and reached 75% at 90 days of ensiling. All oat silages, regardless of the addition of the inoculant, had greater relative abundance of *Enterobacteriaceae* at the 1st day of ensiling, indicating that the LAB started the fermentation of oat silage after 1 day of ensiling. During the first 6 days of ensiling, the relative abundance of *Pediococcus*, possessing high acidification potential, was greater in the LI1-inoculated silages. As a result, there was a rapid decrease in pH of LI1-inoculated silages, which might have restricted plant and microbial proteolytic activity, thereby leading to a lower ammonia N concentration. Cai (43) reported that during the ensiling process, lactic acid-producing cocci, e.g., *Enterococcus*, *Lactococcus*, and *Pediococcus*, grow actively during the initial period of the ensiling process, while *Lactobacillus* is the major LAB during the later stages of ensiling. This might explain the higher relative abundance of *Pediococcus*, *Lactococcus*, and *Enterobacteriaceae* (including *Enterococcus*) in early stage of ensiling. Our study indicated that *Pediococcus* had a negative correlation with DM loss but a positive correlation with LA in LI1. Inoculation of Pediococcus acidilactici enhanced lactate production and DM recovery. Therefore, LAB exert microbial synergy to form a dominant community, effectively inhibiting the growth of undesirable microbes, gas production, and fermentation loss.

The silage fermentation process is regulated by microbial activities via complex metabolic pathways to decompose substrates or shift metabolites (37). The predicted function profiles of bacterial communities in silage enable us to obtain more information about the metabolic pathways of the bacterial community affecting silage quality. Generally, most metabolic categories strengthened with the fermentation progress, indicating that the metabolic capacity of the microbial community was augmented with increasing microbial abundance and diversity (44). Therefore, it is meaningful and necessary to determine metabolic pathways of the bacteria in relation to the oat silage. Figure 5 demonstrates the predicted functional profiles of the bacterial community of control and LAB inoculated silages at different ensiling durations in level 2, determined by analysis of the KEGG databases by PICRUSt2. In this study, among 12 metabolic functions with high abundance, three pathways—global and overview maps, carbohydrate metabolism, and amino acid metabolism—play the predominant role in all silages at different days of ensiling. This suggested that three types of metabolism were essential for microbial metabolism. The pathway of amino acid metabolism represents an ability to convert the macromolecular proteins in forage into peptide substances or amino acids that can easily be taken in by the animals (45). In this study, the lowest amino acid metabolism in the LI1-treated silage at the initial stage of fermentation (1, 3 and 6 days) was consistent with lower pH and ammonia N. Therefore, this result further illustrated that the lowest pH restrained the amino acid metabolism related to Enterobacter in the LI1-inoculated silage (46). In general, the metabolic pathways of glycolysis, pyruvate, and butanoate, which are included in carbohydrate metabolism, may have a substantial impact on palatability of silages for herbivores (45). A higher relative abundance of carbohydrate metabolism was found in LI1-treated silages at 3 days of ensiling. Accordingly, a higher relative abundance of all LAB in the bacterial communities was also found in the LI1-treated silage than in the LI2 silage. Conversely, a higher relative abundance of carbohydrate metabolism was found in LI2 silage at 6 days of ensiling; meanwhile, the relative abundance of total LAB was higher in its bacterial community. This strongly demonstrates that the relative abundance of the LAB community was related to the expression of the carbohydrate metabolism pathway. Few metabolic pathways with significant differences were observed at the end period of fermentation relative to the early stage, which may reflect stable and undifferentiated metabolism by the dominant bacterial community. These findings show that LI1-treated silage improved silage fermentation and reduced protein decomposition compared to LI2-treated silage.

### Conclusions.

The community synergy of LAB and cleaner fermentation of oat silage prepared with a multispecies microbial inoculant were studied. LAB exert microbial synergy to form a dominant community during ensiling, effectively inhibiting the growth of harmful microorganisms, gas production, and fermentation loss. The combined inoculation of *Lactiplantibacillus*, *Lentilactobacillus*, and Pediococcus acidilactici species effectively enhanced the carbohydrate metabolism pathway but inhibited the amino acid metabolic pathway, resulting in improved fermentation quality and reduced protein decomposition of oat silage. The results indicate that oat silage with the L11 inoculant can be used to prepare high-quality, cleaner fermented feed and is a potential feed resource for ruminants.

## MATERIALS AND METHODS

### Microbial inoculants.

Two commercial multispecies inoculants, LI1 (Bonsilage containing Lentilactobacillus buchneri, *Lactiplantibacillus plantarum*, *Lacticaseibacillus paracasei*, and Pediococcus acidilactici; Schaumann Agricultural Trading Co., Ltd., Shanghai, China) and LI2 (Zhuanglemei IV containing Lentilactobacillus buchneri and *Lactiplantibacillus plantarum*; Sichuan Gaofu Ji Biotechnology Co., Ltd., Chengdu, China), were utilized for the silage preparation of oat. The viable counts of LAB in inoculants were ≥1.0 × 10^11^ CFU/g for LI1 and ≥1.3 × 10^10^ CFU/g for LI2.

### Silage preparation.

Oat (*Avena sativa* L. cultivar Qinghai444) was cultivated in an experimental field of the Inner Mongolia Academy of Agricultural and Animal Husbandry Sciences (40°46.265 N, 111°39.851 E; altitude, 1,056 m), located at the foot of Yinshan Mountain on the Tumochuan plain in Inner Mongolia, China. The crop was reaped at the milk stage with approximately 300 g/kg DM on 24 July 2021 and immediately chopped into 1- to 2-cm lengths. Silages were prepared with a laboratory fermentation system (47), and the treatments were designated control, LI1, and LI2. After homogenization of the material, LAB inoculants LI1 and LI2 were processed to 2 g/t and 10 g/t based on FM (fresh matter), respectively. The inoculant was dissolved in sterile distilled water according to the recommended dosage and sprayed with a sprayer. The control was added with an equal amount of sterilized water. After the materials were mixed evenly, 500 g of fresh material was packed in plastic bags (food grade; 300 mm by 400 mm; Anxi County Chengxiang Fengdu Machine Processing Co., Ltd., Quanzhou, China), and the bags were sealed quickly with a sealer (Xiawei ZK-420; Anxi County Chengxiang Fengdu Machine Processing Co., Ltd., Quanzhou, China). For each treatment, 24 bags of silage were prepared and stored at 22°C to 28°C. After 1, 3, 6, 15, 35, and 90 days of ensiling, four bags of silage were randomly selected from each treatment for analysis of microbial community and fermentation parameters.

### Fermentation characteristics and chemical composition analyses.

For analysis of silage fermentation, 180 mL of distilled water was added to 20 g of silage sample, and the mixture was immediately homogenized in a homogenizer (LW-09; Shanghai Jingxin Industrial Development Co., Ltd., Shanghai, China) for 10 min and then filtered through 4 layers of cheesecloth. Extracts of sample were used for pH measurement with a pH meter (PB-10; Sartorius, Gottingen, Germany). Then, the extract sample was filtered through a 0.22-mm filter for analysis of organic acids and ammonium nitrogen by high-pressure liquid chromatography (HPLC) and the Kjeltech autodistillation system (2200; Foss Tecator, Hoganas, Sweden) as described by Cai (48). For microbial population analysis, 20 g of silage sample and 180 mL of 0.85% sterile saline were placed in a sterile beaker and mixed well; then, the mixture was diluted 10^1^ to 10^9^ with a sterile test tube. An MRS agar plate, nutrient agar plate, violet red bile agar plate, and potato dextrose agar plate were used to incubate the LAB, aerobic bacteria, coliform bacteria, and yeasts, respectively, as described by Cai et al. (47). The counts of microorganism were expressed as CFU per gram of FM. For chemical analysis, the silage samples were dried in a ventilated heating dryer at 65°C for 48 h to detect the dry matter. The DM loss of silage was calculated using the difference in DM (by weight) before and after fermentation. The WSC content was determined based on the anthrone-sulfuric acid colorimetry (49). LBC was measured following the method of Playne and McDonald (50). The total N content was determined using a Kjeltech 8400 autoanalyzer (Foss Co., Ltd., Hillerød, Denmark), and CP content was computed by multiplying total N by 6.25. The contents of ADF and NDF were quantified as recommended by Van Soest et al. (51).

### Gas production analyses.

The gas volume in silage bags was measured in a 5,000-mL measuring cup kept in a constant-temperature water bath (25°C) as described by Chen et al. (18). The difference in expansion volume before and after ensiling was calculated as the gas production.

### DNA extraction and 16S rRNA gene sequencing.

DNA extraction and 16S rRNA gene sequencing data processing were performed by LC-Bio Technology Co., Ltd. (Hang Zhou, Zhejiang Province, China). Briefly, the library quantification kit for Illumina (Kapa Biosciences, Woburn, MA, USA) was used to assess the size and quantity of the amplicon. Then, the NovaSeq PE250 platform was used to sequence the library. Alpha diversity was analyzed by QIIME (version 1.9.1), and the relative abundances of microbial communities were measured at the phylum and genus levels using the NT-16S with a confidence threshold of 70%. Bacterial function prediction was proof checked from the KEGG database using phylogenetic investigation of communities by reconstruction of unobserved states (PICRUSt2 v2.3.0_b).

### Statistical analysis.

All the reported results are the means from four replicates. Chemical composition, fermentation quality, alpha diversity, and microbial count data were analyzed using two-way analysis of variance (ANOVA) with Duncan’s multiple-range test and with silage inoculant and ensiling time as the main variables by SAS version 9.1 (SAS Institute, Cary, NC, USA). Differences between results were considered statistically significant when the *P* value was lower than 0.05. One-way ANOVA bar plots were made to demonstrate which genera contributed to the changes in the bacterial community. The relationships between the bacterial taxonomic profile and silage quality variables were determined by calculating the Pearson correlation coefficients and were plotted by using the online Majorbio Cloud platform (www.majorbio.com).

### Data availability.

Raw sequencing files and associated metadata have been deposited in NCBI’s Sequence Read Archive (SRP410438).
